# Supplementary material for: Persistent homology analysis distinguishes pathological bone microstructure in non-linear microscopy images
Source: Sci Rep. 2023 Feb 13;13:2522. doi: 10.1038/s41598-023-28985-3 (PMC9925777; doi:10.1038/s41598-023-28985-3)
Supplement: Supplementary file 1 — Supplementary Figures. [file 41598_2023_28985_MOESM1_ESM.pdf]

Persistent homology analysis distinguishes pathological bone microstructure in non-linear microscopy images: Supplemental Information

Ysanne Pritchard[1,*], **Aikta Sharma**[4,6], **Claire Clarkin**[4], **Helen Ogden**[1,3,5], **Sumeet Mahajan**[2,3,+], **and Rubén J. Sánchez-García**[1,3,5,+]

[1]School of Mathematical Sciences, University of Southampton, Southampton, SO17 1BJ, UK
[2]School of Chemistry, University of Southampton, School of Chemistry, Southampton, SO17 1BJ, UK
[3]Institute for Life Sciences, University of Southampton, Southampton, SO17 1BJ, UK
[4]School of Biological Sciences, University of Southampton, Southampton, SO17 1BJ, UK
[5]The Alan Turing Institute, London, NW1 2DB, UK
[6]Mechanical Engineering, University College London, London, WC1E 7JE, UK
*ysanne.pritchard@soton.ac.uk
+these authors contributed equally to this work

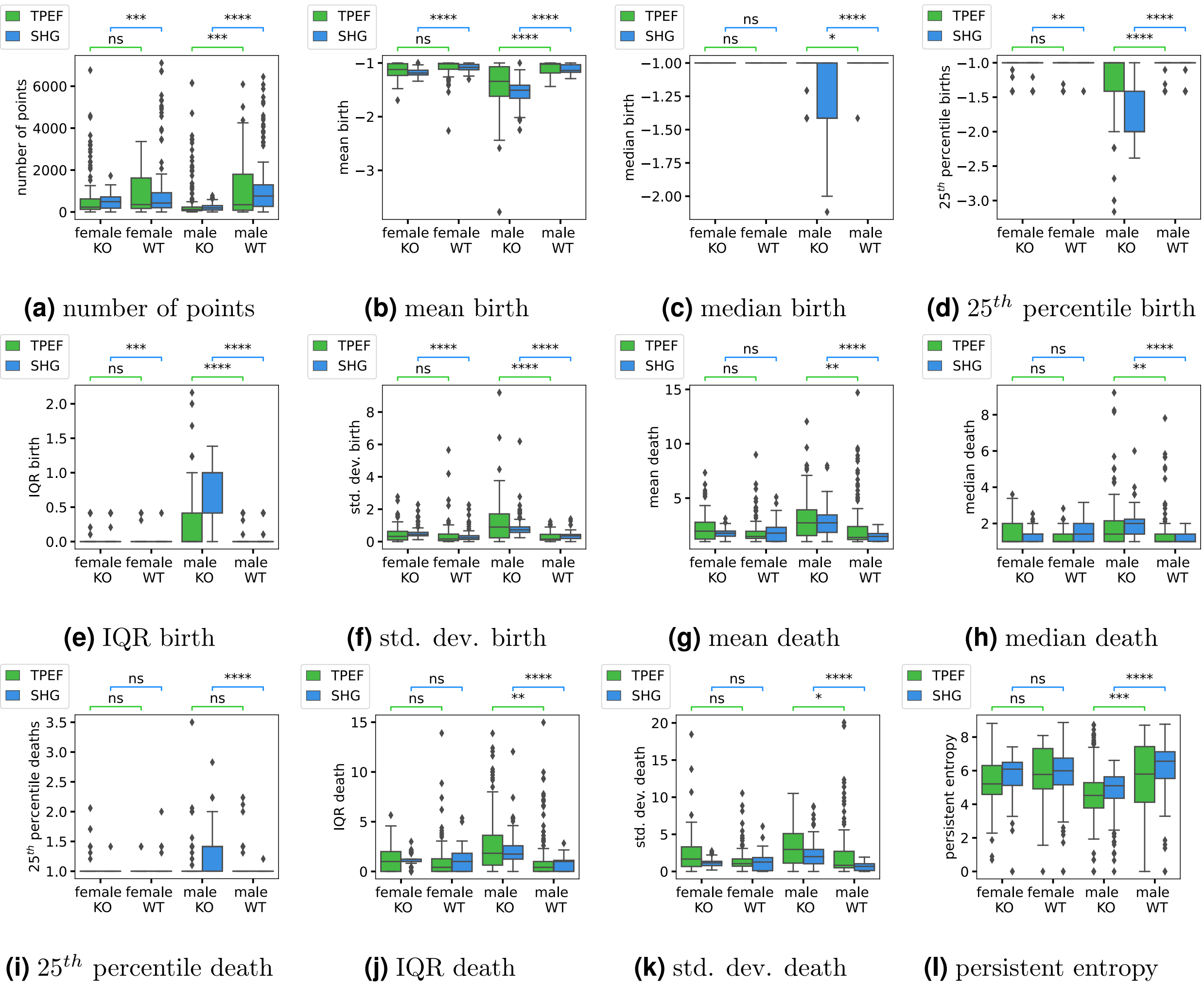

(a) number of points **(b)** mean birth **(c)** median birth **(d)** 25th percentile birth

(e) IQR birth **(f)** std. dev. birth **(g)** mean death **(h)** median death

(i) 25th percentile death **(j)** IQR death **(k)** std. dev. death **(l)** persistent entropy

Figure S1. Box plots of persistence statistics from quadrant 2 of H_0 per image patch, which summarise the micro-holes, including (a) number of points, (b) mean birth, (c) median birth, (d) 25th percentile birth, (e) interquartile range (IQR) birth, (f) standard deviation birth, (g) mean death, (h) median death, (i) 25th percentile death, (j) IQR death, (k) standard deviation death, and (l) persistent entropy. The box plots show the median and quartiles, with outliers marked as points outside of 1.5 times the interquartile range. We annotate their significance using the adjusted p-values for four comparisons (male OcnVEGFKO v. male WT, female OcnVEGFKO v. female WT, male OcnVEGFKO v. male WT, female OcnVEGFKO v. female WT) on each imaging type (TPaF or SHG). Here the stars indicate significance levels: 'ns' is not significant $0.05 < p \leq 1$, '*' $0.01 < p \leq 0.05$, '**' $0.001 < p \leq 0.01$, '***' $0.0001 < p \leq 0.001$, and '****' $p \leq 0.0001$. We include only four of eight significance annotations for readability. Std. dev. is the standard deviation and IQR is the interquartile range.

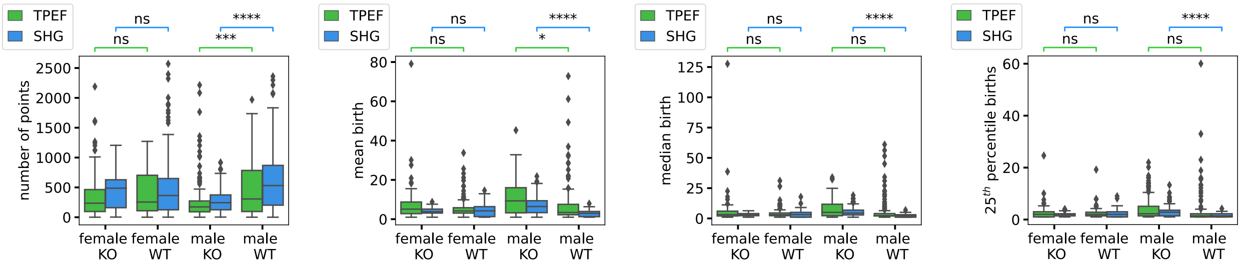

(a) number of loops **(b)** mean birth **(c)** median birth **(d)** 25th percentile birth

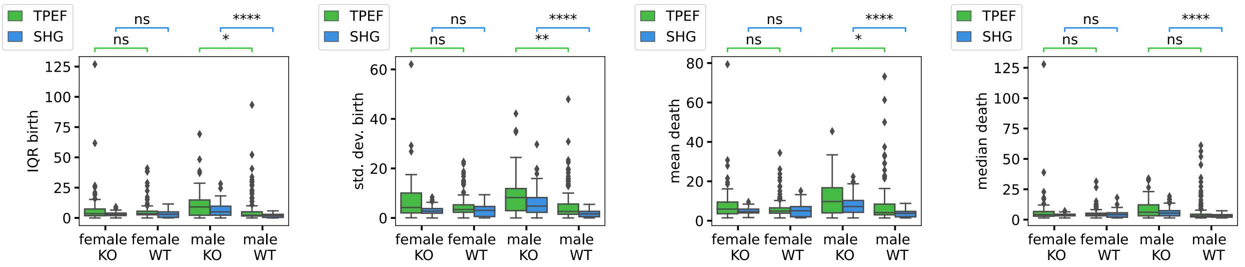

(e) IQR birth **(f)** std. dev. birth **(g)** mean death **(h)** median death

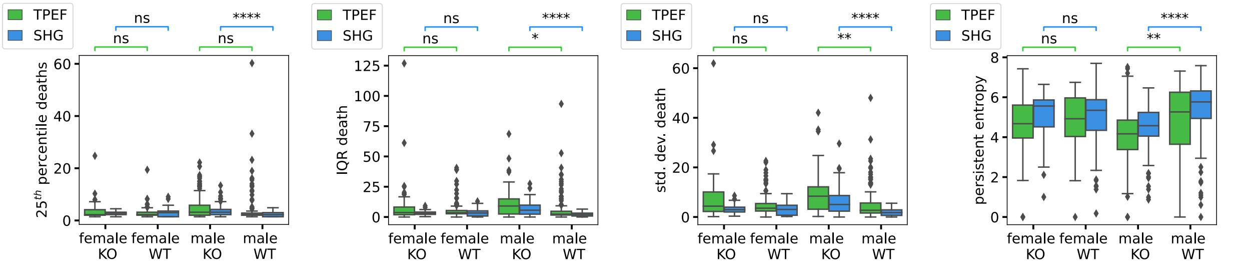

(i) 25th percentile death **(j)** IQR death **(k)** std. dev. death **(l)** persistent entropy

Figure S2. Box plots of persistence statistics from quadrant 1 of H_1 per image patch, which summarise the loops in the filtration that are regions of bone surrounded by micro-holes, including (a) number of loops, (b) mean birth, (c) median birth, (d) 25th percentile birth, (e) interquartile range (IQR) birth, (f) standard deviation birth, (g) mean death, (h) median death, (i) 25th percentile death, (j) IQR death, (k) standard deviation death, and (l) persistent entropy. The box plots show the median and quartiles, with outliers marked as points outside of 1.5 times the interquartile range. We annotate their significance using the adjusted p-values for four comparisons (male OcnVEGFKO v. male WT, female OcnVEGFKO v. female WT, male OcnVEGFKO v. male WT, female OcnVEGFKO v. female WT) on each imaging type (TPaF or SHG). Here the stars indicate significance levels: 'ns' is not significant $0.05 < p \leq 1$, '*' $0.01 < p \leq 0.05$, '**' $0.001 < p \leq 0.01$, '***' $0.0001 < p \leq 0.001$, and '****' $p \leq 0.0001$. We include only four of eight significance annotations for readability. Std. dev. is the standard deviation and IQR is the interquartile range.
